# Phosphorus Co-Existing in Water: A New Mechanism to Boost Boron Removal by Calcined Oyster Shell Powder

**DOI:** 10.3390/molecules27010054

**Published:** 2021-12-22

**Authors:** Chi-Hao Yang-Zhou, Jia-Xin Cao, Shan-Shan Dong, Su-Hua Chen, Ruby N. Michael

**Affiliations:** 1 Key Laboratory of Jiangxi Province for Persistent Pollutants Control and Resources Recycle, Nanchang Hangkong University, Nanchang 330063, China; 1902085229145@stu.nchu.edu.cn (C.-H.Y.-Z.); 2002085700174@stu.nchu.edu.cn (J.-X.C.); 71048@nchu.edu.cn (S.-S.D.); 2 School of Engineering and Built Environment, Griffith University, Nathan, QLD 4111, Australia; ruby.michael@griffith.edu.au

**Keywords:** waste recycling, simultaneous removal, HAP, occlusion co-precipitation

## Abstract

The removal of boron (B) from water by co-precipitation with hydroxyapatite (HAP) has been extensively studied due to its low cost, ease of use and high efficiency. However, there is no explicit mechanism to express how resolved B was trapped by HAP. Thus, in this work, the process of removing B from water was studied using a low-cost calcium (Ca) precipitation agent derived from used waste oyster shells. The results showed that the removal rate of B in the simulated wastewater by calcined oyster shell (COS) in the presence of phosphorus (P) is up to more than 90%, as opposed to virtually no removal without phosphate. For B removal, the treated water needs to be an alkaline solution with a high pH above 12, where B is removed as [CaB(OH)_4_]^+^ but is not molecular. Finally, the synergistic mechanism of co-precipitation between HAP and dissolved B, occlusion co-precipitation, was explained in detail. The proposed method discovered the relationship between Ca, P and B, and was aimed at removing B without secondary pollution through co-precipitation.

## 1. Introduction

Boron (B) is a pervasive non-metallic element naturally present in combination with oxygen in surface water and groundwater. B concentrations in surface water range widely from 0.001 to 150 mg/L, usually in the form of boric acid, and are not reduced by the treatment of drinking water [1]. As a result, ingestion of B from water is one of the most common routes of human environmental exposure. Although B has been recognized as an essential nutrient for plants and animals, recent exposure to B has been implicated as a cause of acute kidney damage and a potential cause of chronic kidney disease in southeast Asia. Therefore, the World Health Organization has formulated a guideline value of 2.4 mg/L B for drinking water, while individual countries have implemented stricter standards [2]. In fact, soluble B can more often be detected at low to medium concentrations due to the industrial release of B-containing pollutants and wastewater from borosilicate glass, cosmetics, textiles, paints, wood processing, detergents, insecticides, drugs, and moderators in nuclear reactors [3]. The interval where B is converted from a nutrient to a contaminant is fairly narrow. In order to prevent the harmful effects of B on biota, great efforts have been made to reduce B residues in the water [4]. 

Adsorption and chemical precipitation are mainly applied to recover B from water [5,6]. The adsorption method involves chelation reactions and electrostatic attraction. In this procedure, boric acid is first grafted covalently onto an absorbent by means of a B-hydroxyl couple, which finally performs immobilization on the materials. In addition, boric acid is a weak acid that not only easily combines with hydroxyl groups to form ionic states which can be removed by positive electric attraction, but also complexes with metal ions to form positively charged ion groups to be fixed by negatively charged materials. However, the main problem encountered in recovering B via adsorption is the requirement of low B-containing streams and a high dose of metal salts. The chemical precipitation method is designed to generate B and calcium (Ca) compounds, which is considered the best choice to remove high concentrations of pollution [7,8]. Unfortunately, additional coagulants are required because the precipitates formed during the process settle very slowly, resulting in high costs and a complex operation. Additionally, the low removal efficiency of B has been observed with chemical precipitation at low B concentrations [9]. Based on these conditions, the co-precipitation of B at a wide range of concentrations with hydroxyapatite (HAP) was investigated using Ca(OH)_2_ mineralized in the presence of P [10,11,12]. During the formation of HAP, the B dissolved in an aqueous solution deposited at the surface of HAP, which can be readily separated from the solution. The removal mechanism is mainly attributed to the combination of B and metal ions in HAP. However, this has been considered questionable because HAP is a good bio-porous material which can be substituted by heavy metal ions for Ca ions, oxyacid for phosphate ions, and halide ions for hydroxyl groups. The formation of HAP requires an excess of Ca, typically in the form of Ca(OH)_2_, as compared to the molar ratio of Ca and P in HAP [13,14]. Cost-effective and easily attainable Ca sources must be considered as an ideal approach to co-precipitation to retrieve B from solutions.

It is well known that oyster shells contain substantial amounts of CaCO_3_ that can be converted into CaO or Ca(OH)_2_ [15,16,17,18]. However, despite this resource, the shells are generally considered waste residues in need of disposal due to their undesirable odour and unpleasant appearance [19,20]. The reuse of oyster shells in environmental applications therefore offers the potential to derive value from this waste stream and reduce the cost of environmental remediation. In this study, the removal of B was examined through using oyster shell powder as a source of Ca and as a mineralizer, and the influence of HAP, Ca and pH were systematically investigated. A reasonable mechanism for B removal by HAP, namely occlusion co-precipitation, was confirmed by our experiments and characterization.

## 2. Materials and Methods

### 2.1. Materials

The waste oyster shells used in this study were gathered from a local market in Nanchang, Jiangxi Province, China. The shells were rinsed with tap water to remove attached sediment and air-dried. All chemicals from commercial sources were of analytical grade and used as received and were purchased from Xilong Scientific, China. Deionized water was used throughout the whole experiment.

### 2.2. Preparation of Modified Oyster Shell Powder

The oyster shell was mechanically crushed and pulverized in a grinder to pass through a 200 mesh. The sifted powder was then heated in a muffle furnace under dry air at 900 °C for 3 h. The calcined oyster shell powder is referred to as COS hereafter.

### 2.3. Set-Up of Ca, P and B Reaction Systems

#### 2.3.1. B Removal with P Addition

To investigate the specific mechanism of B removal, NH_4_H_2_PO_4_, which showed the best kinetic performance in P species for B removal, was selected as a co-precipitation reagent [3]. In general, 0.3434 g COS as a Ca source and an alkaline reagent were added to 100 mL of solution containing 142.76 mg/L H_3_BO_3_ (25 mg/L B) and 2.12 g/L NH_4_H_2_PO_4_ (570 mg/L P). The initial pH of the mixture was then adjusted to 3, 5, 7, 9 and 12 respectively, utilizing 1 mol/L NaOH and HCl. For comparison, only COS and 100 mL of 25 mg/L B was mixed followed by a pH adjustment. The resulting mixture was shaken horizontally at 180 rpm for 24 h on a rotary shaker at 25 °C until it was balanced. After an ageing period of 24 h, the supernatants were separated by centrifugal filtration, and the collected precipitates were washed several times with deionized water and dried at 60 °C for 12 h. The supernatant was provided to determine the pH by a pH meter and the concentration of residual B and P in water by visible spectrophotometry using the azomethine H method and the ammonium molybdate method, respectively [21].

#### 2.3.2. Optimum Ca/P Molar Ration for Co-Precipitation Experiment

The molar ratio of Ca/P in pure HAP is 5:3. A batch of settling experiments were designed to get information on the precipitation mechanism by adding COS to 100 mL of solutions containing 25 mg/L B and 570 mg/L P to achieve P/Ca molar ratios of 0.15, 0.2, 0.3, 0.4, 0.5 and 0.6 respectively. All resulting mixtures were then subjected to the same balance processes and separation steps as described above.

#### 2.3.3. Adsorption Experiments

Adsorption experiments were carried out with an air bath shaker at 180 rpm. To assess the role of adsorption on B removal, 0.308 g of HAP was used as an adsorbent to remove B from 100 mL of 25 mg/L B solution with pH values of 3, 5, 7, 9 and 11, separately. For comparison, the solid of HAP and CaCl_2_ was mixed with a reaction solution consisting of 25 mg/L B and 570 mg/L P for 24 h. Subsequent separation tests were performed in the same manner as described in Section 2.3.1.

The experiments mentioned above have been carried out at least three times.

### 2.4. Characterization

The crystal structures of the as-prepared COS and collected precipitates were studied using the X-ray diffractometer (XRD, Bruker, model D8ADVANCE, Berlin, Germany) with Cu Kα radiation (=0.15406 nm). Fourier transform infrared spectra (FT-IR, Bruker Optic VERTEX 70, Germany) were collected with wavelengths of 400–4000 cm^−1^. X-ray photoelectron spectroscopy (XPS, Thermo Fisher Scientific, ESCALAB 250, Waltham, MA, USA) analyses of precipitates were carried out in an ultrahigh vacuum chamber. Solid ^11^B-nuclear magnetic resonance (^11^B-NMR, JEOL JNM ECZ600R, JEOL Ltd., Akishima, Japan) spectra of the precipitates which resulted from removing P and B with COS were acquired using 4 nm probes and a single pulse method. The resonance frequency for ^11^B was 192.56 MHz at a field strength of 600 MHz. Typical acquisition parameters were pulse length 0.1 ms and relaxation delay 2 s.

## 3. Results

### 3.1. Occurrence of P on B Removal from Water

**Figure 1:**
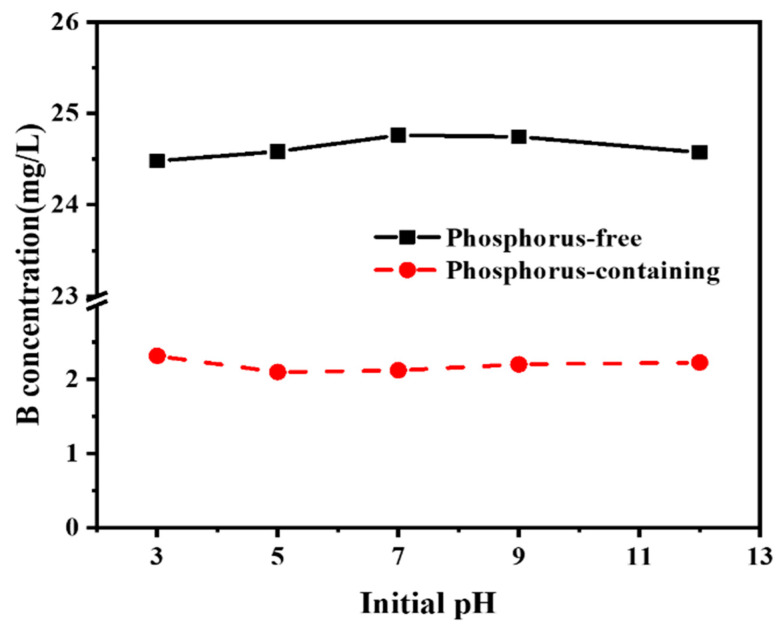
The performance of B removal by calcined oyster shells (COS) singly and in combination with phosphorus (P).

**Figure 2:**
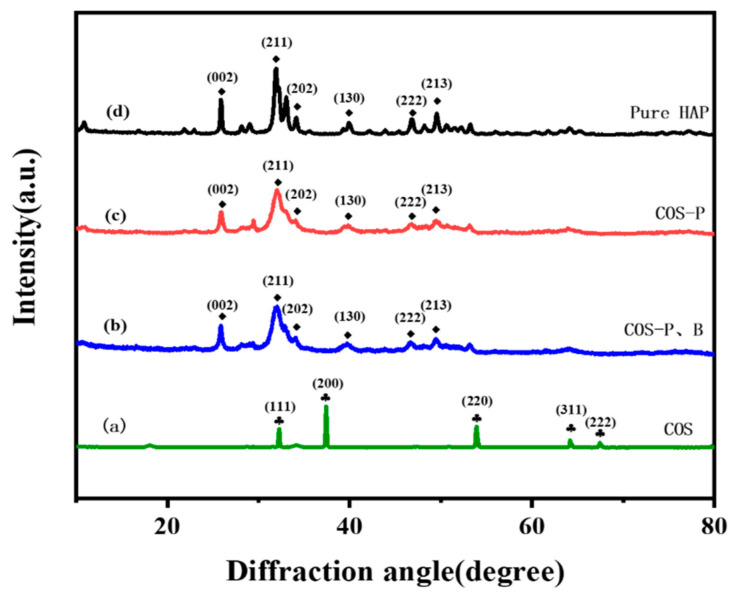
The XRD patterns of material and reactive residual solids (**a**: COS, **b**: COS after B and P reaction, **c**: COS after P and **d**: pure HAP).

The effects of the presence of P in the solution for B removal are illustrated in Figure 1. Obviously, when P occurred in water, the concentration of B decreased dramatically from 25.0 mg/L to around 2.2 mg/L with the addition of COS. Otherwise, almost 98.4% of initial B was retained in water when the water was free of P. Chemically, oyster shell powder is similar to that of chalk, both of which are predominantly CaCO_3_ [22]. After calcination, oyster shell powder was primarily transformed into CaO, which is confirmed by the consistency of diffraction peaks between pure CaO (PDF#37-1497) and COS, as shown in Figure 2a. It is well known that the liquid-solid reaction between water and CaO leads to the formation of Ca(OH)_2_, which has been widely used as a precipitant and alkaline reagent to immobilize P in water [23]. These results indicated that oyster shell powder can be used to replace chalk as a functional reagent to sequester P from water, which has many environmental advantages, including avoiding environmentally damaging and energy-consuming chalk quarries and the reduction of local oyster shell pollution.

Although B removal by means of chemical precipitation with Ca(OH)_2_ has succeeded in the recovery of B from solution [24], the removal efficiency depends greatly on the operational parameters of chemical precipitation. In short, a low pH value, high initial B concentration and high temperature are required to achieve a high B removal efficiency. The solution temperature of 25 °C in this study is too low for the scattering of the borate ions through the external boundary layer and in the internal pores of the Ca(OH)_2_ particles to form calcium borate species. As Alper Erdem Yilmaz stated, an increase in temperature will accelerate the equilibrium capacity of the Ca(OH)_2_ for particular borate ions; thus, a B recovery efficiency of above 90% of can be obtained at a temperature of 90 °C within 2 h, while a recovery efficiency of only 30% can be obtained at 20 °C for 1000 mg/L of the initial B concentration. Therefore, chemical precipitation with the Ca(OH)_2_ mineralizer for B removal from the aqueous solution is not an efficient technique at low temperature.

In the case of P addition, high B recovery efficiency may be attributed to the formation of HAP, which involves two steps: nucleation and crystal growth. First, a base was formed on which the nucleation of the heterogeneous intermediate takes place. This was followed by the growth of the monocrystalline HAP. Hydrothermal transformations of biogenetic CaO to HAP can be illustrated as Equations (1) and (2).

(1) $${\mathrm{CaO}+H}_{2}{O=\mathrm{Ca}(\mathrm{OH})}_{2}$$

(2) $${10\mathrm{Ca}}^{2+}{+6\mathrm{PO}}_{4}^{3-}{+2\mathrm{OH}}^{-}⇌{\mathrm{Ca}}_{10}{\left({\mathrm{PO}}_{4}\right)}_{6}{\left(\mathrm{OH}\right)}_{2}$$

In this synthesis, octa-calcium phosphate (OCP) transferred rapidly to amorphous calcium phosphate (ACP), and subsequently from ACP to calcium-deficient HAP (DAP) and HAP [25]. The formation of HAP was verified by the diffraction pattern, which was a feature of HAP crystal orientation within the sample of the collected solid precipitate (Figure 2b–d). Furthermore, the characteristics of HAP phase were reserved as pure HAP, irrespective of the presence or absence of B in the solution, which means that the introduction of B causes no change in the lattice parameters of HAP or in the morphology of B-containing phases [26]. The results indicated that the borate ions were not removed by replacing the phosphate radical of precipitates in HAP [27].

### 3.2. B Removal through Co-Precipitation

**Figure 3:**
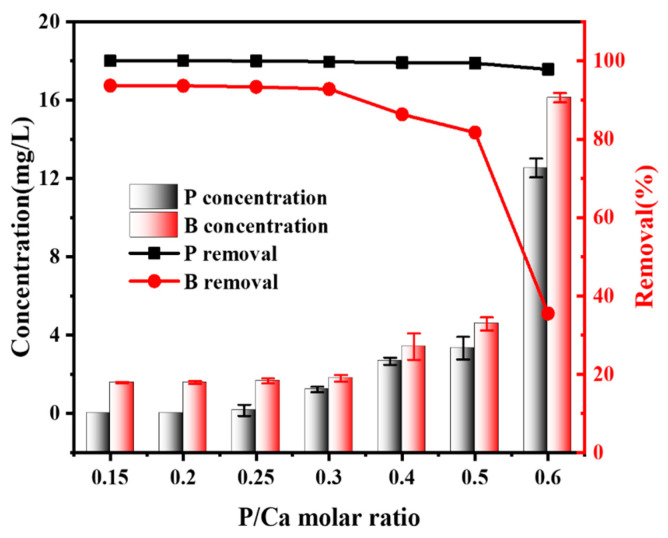
Removal performance of different rations of P/Ca using COS.

As noted above, removal of B by the formation of HAP has been observed, and the effects of molar ratios of P/Ca on B removal from solution during HAP precipitation are illustrated in Figure 3. The amount of residual B and P in solution increased rapidly as the molar ratio of P/Ca increased from 0.3 to 0.6, as the Ca(OH)_2_ produced during the resolution of COS in water was slightly soluble. This situation decreased the reaction rate between Ca^2+^ and PO_4_^3−^, resulting in excessive PO_4_^3−^ that could not be precipitated as HAP. From the point of view of equilibrium of the precipitation reaction, the K_sp_ for HAP at 25 °C is 1 × 10^−126^, which means that an incomplete reaction will occur when the molar ratio of P/Ca is 0.6 and residual P will be detected due to the lack of Ca^2+^ to precipitate P completely. When the molar ratio of P/Ca is below 0.3, the concentration of soluble Ca^2+^ of Ca(OH)_2_ is large enough to almost completely precipitate all of the PO_4_^3−^ in the solution, with a removal rate above 99.79%.

Interestingly, the trend of residual B in solution with the changing molar ratio of P/Ca was similar to that of residual PO_4_^3−^_._ It is known that the energy required to combine PO_4_^3−^ and Ca^2+^ is lower than that of BO_3_^3−^ and Ca^2+^, so Ca^2+^ was predominantly combined with PO_4_^3−^ and partially combined with BO_3_^3−^ [28,29]. This was likely the reason why the removal efficiency of B became lower when the content of Ca^2+^ decreased, and vice versa [25,26]. When the molar ratio of P/Ca was below 0.3, soluble Ca^2+^ from Ca(OH)_2_ was enough to precipitate both PO_4_^3−^ and BO_3_^3−^ to obtain acceptable concentrations of PO_4_^3−^ and BO_3_^3−^, but an excess amount of Ca^2+^ in solution combined with a large consumption of COS are challenges for water quality management and water treatment economics. Thus, the molar ratio between PO_4_^3−^ and Ca^2+^ of 0.3 is the best choice to succeed in both removing BO_3_^3−^ from solution to an acceptable concentration and to avoid secondary pollution.

Therefore, it can be inferred that the removal of B was related to the formation of HAP. The results of the P/Ca ratio affecting the formation of HAP were similar to those reported before, and the best option for the P/Ca molar ratio is 0.3 [30]. However, COS instead of Ca(OH)_2_ or CaCO_3_ can drastically reduce the consumption of reagent as well as create mild and easy reaction conditions [31]. Overall, we propose that the interaction between B, P and Ca contributes to the HAP formation process with the removal of B.

### 3.3. Adsorption Mechanism

**Figure 4:**
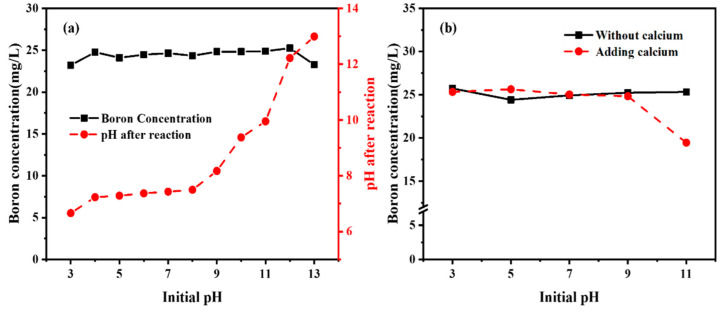
Removal performance and pH (**a**) pure hydroxyapatite (HAP); (**b**) HAP and extra calcium.

HAP is widely used in adsorption because it is characterized as a porous, thermally stable, non-toxic, and inexpensive medium. So far, no report has been published on the direct adsorption of borate by HAP, but organics containing hydroxyl groups have usually been applied to modify the surface of membranes to complex with B [5]. To investigate the contributions of abundant hydroxyl groups in HAP to the removal of B in solution, the direct adsorption of B by HAP was studied. The results are shown in Figure 4a. No obvious effect on the removal of B was observed by HAP in either acidic or alkaline solutions. The HAP/water interface can be described by a chemical model considering two different types of surface groups: positively charged CaOH_2_^+^ surface species and negatively charged PO^4−^ surface species. The surface of HAP was negatively charged in alkaline solutions due to the presence of PO_4_^−^, which means that borate ions cannot be attracted to the surface of HAP [32,33]. On the other hand, positively charged CaOH_2_^+^ species predominate in acidic solutions, making the surface charge of HAP positive in this pH range, but borate ions were not electrostatically attracted to the surface of the HAP even if the surface was positively charged under acidic conditions. These results indicated that the HAP has little effect on the removal of B, and this was assumed to be because the HAP is relatively stable and does not easily release large amounts of Ca ions.

In addition, the residual B concentration in the solution decreased with the increase in Ca addition, as shown in Figure 3, and thus, Ca^2+^ can be an influencing factor in the removal of B. To explore why it plays a role in removing B, CaCl_2_ was added to the HAP reaction system. The borate concentration decreased slightly when the pH exceeded 7, especially at 13, with a removal efficiency of about 13.65% (Figure 4b). The results were strikingly similar to the study of B adsorption by MgO, which might be due to the large amount of Ca^2+^ attracting borate ions and composing them in a 1:1 ratio to form groups of charged ions as in Equations (3) and (4). The results demonstrated that [CaB(OH)_4_]^+^ were electrostatically attracted and adsorbed by negatively charged adsorbents in solution, which is the actual reason why B can be removed by HAP precipitation [34,35].

(3) $$H_{3}{\mathrm{BO}}_{3}{+\mathrm{OH}}^{-}⇌B{\left(\mathrm{OH}\right)}_{4}^{-}$$

(4) $${\mathrm{Ca}}^{2+}+B{\left(\mathrm{OH}\right)}_{4}^{-}⇌{\left[{\mathrm{CaB}(\mathrm{OH})}_{4}\right]}^{+}$$

**Figure 5:**
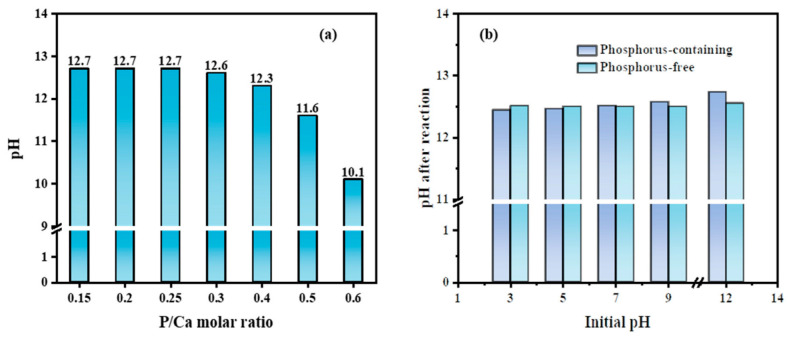
The pH of (**a**) P/Ca and (**b**) P i006E solution after reaction.

The principal component of COS was CaO, which dissolved in water to form Ca(OH)_2_, resulting in an alkaline solution. The pH after the reaction in various molar ratios of P/Ca and in the presence or absence of P is displayed in Figure 5. The pH was practically unchanged when the ratio was above 0.3, and all solutions were alkaline after precipitation (Figure 5a). Moreover, there was essentially no change in pH after the reaction, regardless of whether P was present (Figure 5b). The initial pH value of the solution was of little importance; it was still alkaline after the reaction if COS was used as a source of Ca^2+^ to precipitate the P.

**Figure 6:**
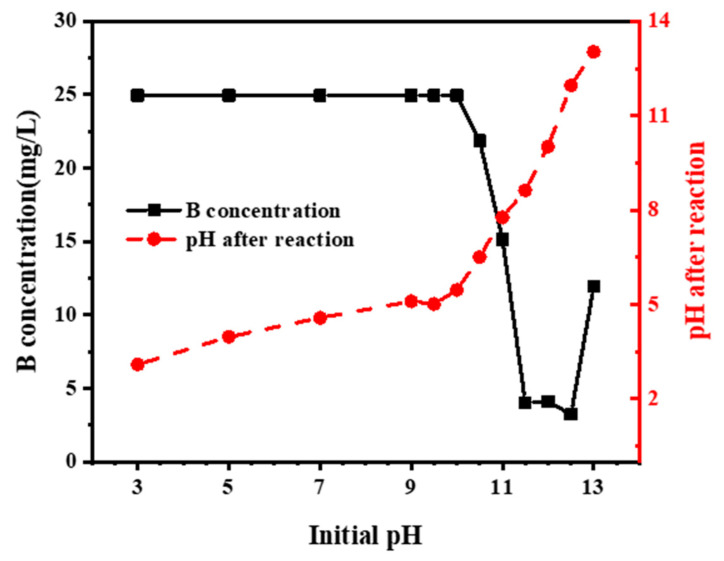
B removal capacity of 61.3 mmol CaCl_2_ at different pH.

To study the influence of the pH of the solution on the removal of B, acidic CaCl_2_ instead of COS was used as a Ca^2+^ source to precipitate P and remove B from solution. Removal efficiency increased as pH increased from 10 to 12.5, and peaked at pH 12.5, followed by a rapid decrease as pH increased (Figure 6). Dissolved B will occur in two different forms: the molecule [B(OH)_3_] and its conjugated base, the borate ion [B(OH)_4_^−^] [36,37]. Their predominance in the aqueous solution usually varied with pH. As the pH of the solution increased, the concentration of [B(OH)_4_^−^] increased and [B(OH)_3_] decreased. B(OH)_4_^−^ begins to appear at a pH of 6 and when the pH is 12.5, all boron is present as [B(OH)_4_^−^] in solution. Therefore, the initial equilibrium of Eq. (4) cannot be maintained and the reaction between Ca^2+^ and B(OH)_4_^−^ happened to a greater extent to form more [CaB(OH)_4_]^+^, which is easily attracted and adsorbed by HAP into solution. In addition, the pH will affect the surface charge of the material. Therefore, B was only removed under alkaline conditions. Contrary to this, it can be seen that B removal is related to the pH after the reaction rather than the initial pH of the solution. 

### 3.4. Characterization Analysis

**Figure 7:**
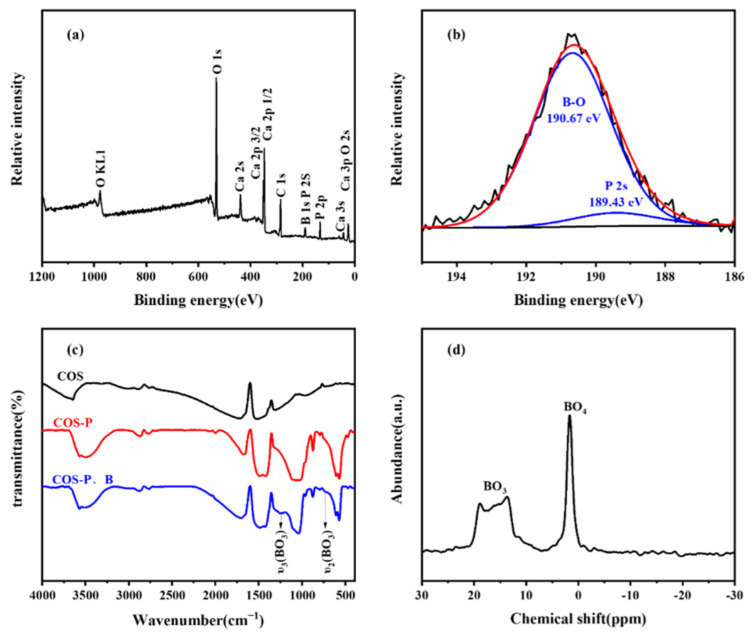
XPS wide spectrum (**a**), XPS spectra of P (**b**), FT-IR spectra (**c**), and ^11^B-NMR spectrum (**d**) of COS after reaction.

Upon learning about the potential mechanism of removal of B, FT-IR, XPS and NMR was used to characterize the resulting substances from precipitation. Large-scale XPS scans of the COS after adsorption were carried out to show the composition of the elements. As shown in Figure 7a, C, O, and Ca have been identified as the primary elements, and B and P have less strong signals. It was likely that excessive COS dissolves in water to form Ca(OH)_2_ and adsorbs CO_2_ from the air, resulting in a strong peak of C adsorption. The B 1s spectrum was deconvoluted into two peaks after adsorption of B at the binding energies of approximately 190.67 eV and 189.43 eV in Figure 7b, corresponding to the B-O and P 2S bonds [38,39], respectively, according to means of least squares curve fitting. Simultaneous removal is likely to happen because of their similar binding energy.

The XPS spectra of COS before and after the reaction were recorded as shown in Figure 7c, which allowed us to find the presence of the band defined as groups BO_3_^3−^. The bands at 1245 cm^−1^ and 725 cm^−1^ are attributed to the antisymmetric stretching υ_3_ and the symmetric bending υ_2_ of the BO_3_^3−^ groups, respectively, which were consistent with other studies [40,41]. However, the vibration peak intensity was not obvious, and many characteristic peaks of borate coincide with HAP.

Solid-state nuclear magnetism and X-ray photoelectron spectroscopy were further used to confirm the method. The ^11^B-NMR spectra for the solid residues after the equilibrium of co-precipitation were shown with the coordination of B in Figure 7d, which means that B was not simply fixed in the form of [B(OH)_4_^−^]; rather, the structural change occurred after fixation. With an initial B concentration of 2.31 mmol, the peak area ratio of ^3^[B]/^4^[B] is estimated to be 0.74, so borate is mainly fixed in the form of tetragonal borate. The results of solid NMR of the precipitated material were completely different from those of a pure boric acid solid. As a result, it was basically concluded that the boron in the solid is not in the form of borate [42,43]. 

**Figure 8:**
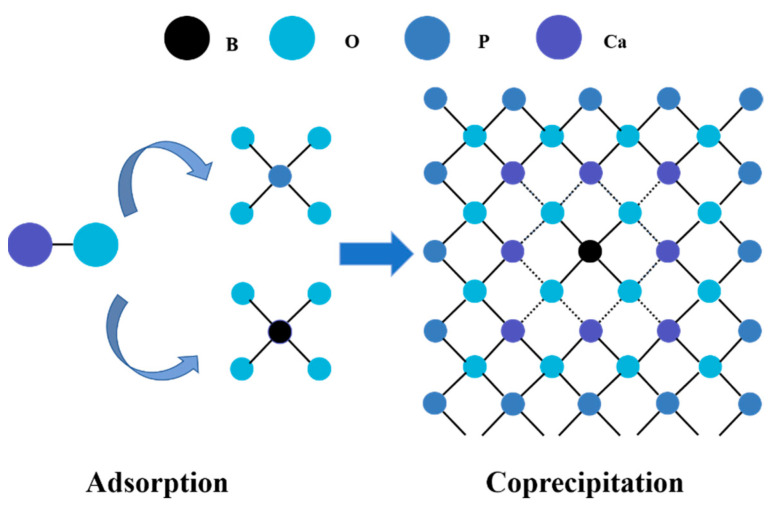
Schematic diagram of B removal mechanism by COS under the intervention of phosphorus (circle in black, light blue, deep blue, and purple represents B, O, P and Ca, respectively).

In comparing the results of the B co-precipitation and adsorption experiments, it was interesting to note that the efficiency of adsorption was not the same as that of co-precipitation. The schematic diagram of the mechanism for B removal was shown in Figure 8. The COS reacts with water to form Ca(OH)_2_ and provides Ca^2+^; it then reacts with the P in the solution to form HAP. Ca^2+^ in the solution combines with borate ions. The formed HAP with negative charge attracts the positive groups of boric acid complexed with Ca to be continuously covered by new forms of HAP, thus achieving a high efficiency of B removal.

## 4. Conclusions

In this study, a method of wastewater treatment using COS combined with P to remove B was investigated and was successful in reducing the concentration of B to an acceptable level. COS is a potential source of Ca to combine PO_4_^3−^ to form HAP and separate the B from the solution. Unlike co-precipitation or adsorption alone, B was not removed as insoluble tiny particles, calcium borate or precipitant of H_3_BO_3_ with HAP, but as negatively absorbed [CaB(OH)_4_]^+^. Occlusion co-precipitation is therefore expected to be the mechanism for effective B removal. The synergistic mechanism of the COS and P for the removal of soluble B has benefits for the water treatment process: it reduces the pressure on biological or membrane treatments and further reduces the concentration of pollutants. It also has broader environmental benefits through the beneficial re-use of oyster shells as a waste product.

## Data Availability

The data used to support the findings of this study are available from the corresponding author upon request.

## Sample Availability

Samples of the compounds are available from the authors.
